# Impact of preoperative nasopharyngeal cultures on surgical site infection after open heart surgery

**DOI:** 10.1016/j.xjon.2021.09.019

**Published:** 2021-09-16

**Authors:** Yoshiyuki Takami, Kentaro Amano, Yusuke Sakurai, Kiyotoshi Akita, Ryosuke Hayashi, Atsuo Maekawa, Yasushi Takagi

**Affiliations:** Department of Cardiovascular Surgery, Fujita Health University School of Medicine, Toyoake, Japan

**Keywords:** surgical site infection, open heart surgery, nasopharyngeal culture, microorganisms, ITA, internal thoracic artery, MRSA, methicillin-resistant *Staphylococcus aureus*, NCx, nasopharyngeal cultures, SSI, surgical site infection, VAC, vacuum-assisted closure

## Abstract

**Objectives:**

Despite advances in surgical techniques and management, surgical site infection (SSI) is still important after cardiovascular surgery. We investigated to determine whether or not preoperative nasopharyngeal cultures (NCx) can predict SSI and its microbial spectrum.

**Methods:**

A retrospective review was done in 1226 consecutive patients undergoing NCx and cardiac and thoracic aortic surgery via median sternotomy who were cared for with the standard SSI bundle between 2013 and 2018. Microorganisms isolated from the NCx and SSI pathogens were counted to explore the microbial pattern and associated variables in patients with and without postoperative SSI. Perioperative management was not changed by collection of preoperative NCx.

**Results:**

There were 1281 and 127 microorganisms, including coagulase-negative *Staphylococcus* as the most prevalent, isolated from 784 nasal and 111 pharyngeal specimens, respectively. Postoperative SSI occurred in 31 patients (2.47%), including chest, groin, and leg SSI. Significant coincidence of the SSI pathogens with the NCx microorganisms was not observed. However, the patients with SSI showed significantly higher positive rates of preoperative NCx than those without SSI. The sensitivity/specificity of NCx for SSI were 81%/37% for nasal and 45%/92% for pharyngeal, respectively. The negative predictive value of NCx for ruling out SSI was 98.6% for nasal and 98.4% for pharyngeal, respectively. Independent risk factors for postoperative SSI included female sex, diabetes mellitus, positive preoperative NCx, and postoperative use of Portex Mini-Trach (Smiths Medical, Minneapolis, Minn) or tracheostomy on multivariate analysis.

**Conclusions:**

Preoperative NCx may be useful to predict SSI after open heart surgery via median sternotomy, as well as screening for methicillin-resistant *Staphylococcus aureus*.

**Figure:**
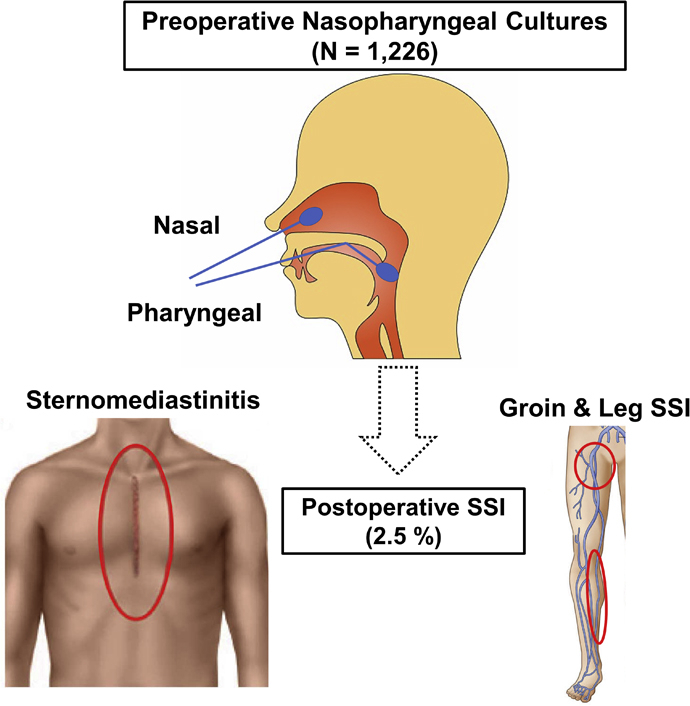
Preoperative NCx to predict SSI after open heart surgery via median sternotomy.

**Central Message**

Positive preoperative nasopharyngeal culture was an independent predictor of SSI after cardiac and thoracic aortic surgery via median sternotomy.

**Perspective**

Although preoperative NCx have been used for MRSA screening, the positive NCx for microorganisms other than MRSA may also be useful to predict SSI after cardiac and thoracic aortic surgery via median sternotomy.

See Commentaries on pages 487 and 489.

Despite advances in surgical techniques and perioperative management, nearly 5% of patients continue to experience major infection after cardiac surgery with increased morbidity, death, and costs.^1^^,^^2^ Among these infections, surgical site infection (SSI) accounts for one-fifth, including mediastinitis and deep incision SSI of the chest and groins. Although the incidence of SSI shows a decreasing trend, it remains stable over time.^3^^,^^4^ Therefore, we should continue to work to eliminate the occurrence of SSI.

Practice guidelines and expert consensus documents recommend screening for nasal carriage of methicillin-resistant *Staphylococcus aureus* (MRSA) and a choice of prophylactic antibiotics.^5^, ^6^, ^7^ However, nasal microorganisms other than MRSA have been rarely studied. Also, the changing of microbiological patterns in the current modern antibiotic era has been little studied. This study investigated to determine whether or not preoperative nasopharyngeal cultures (NCx) are useful to predict SSI and its microbial spectrum in patients undergoing open heart surgery via median sternotomy (Figure 1).

**Figure 1:**
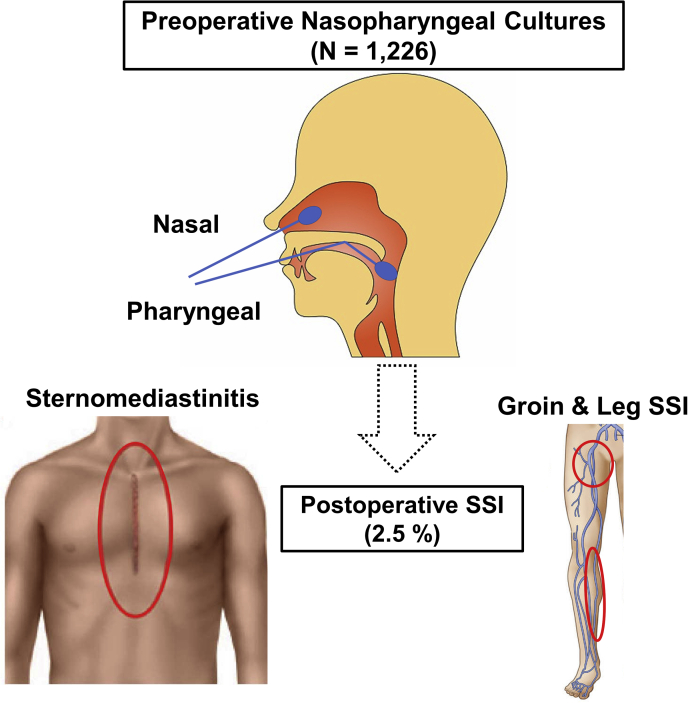
This study investigated to determine whether or not preoperative nasopharyngeal cultures, including screening for carriage of methicillin-resistant *Staphylococcus aureus*, are useful to predict surgical site infection (*SSI*) and its microbial spectrum in patients undergoing cardiac and thoracic aortic surgery via median sternotomy.

## Methods

### Study Patients

This retrospective study was conducted on 1265 consecutive patients undergoing preoperative NCx and cardiac and thoracic aortic surgery via median sternotomy at Fujita Health University Hospital, Toyoake, Japan, from September 2013 to August 2018. We excluded 39 emergency surgery patients who could not undergo preoperative NCx. This retrospective observational study was approved by the institutional research review board (No. HM20-280, September 7, 2020) and individual oral and written informed consent was waived for the retrospective use of patients' data. It was also conducted according to the ethical guidelines for clinical study published by the Ministry of Health and the Helsinki Declaration. Our SSI control measures were carried out mainly according to the SSI bundle consisting of 19 pre-, intra-, and postoperative items by Miyahara and colleagues.^8^ We also referred partially to US^5^ and European^6^ expert consensuses. During the study period there were no chronological changes in preoperative, intraoperative, and postoperative management, except for wound dressings, as follows.

### Preoperative Management and NCx

In all patients, nasal and pharyngeal swabs were obtained for cultures at the outpatient clinic or emergency room before surgery. Microbiology culture and antibiotic sensitivity or resistance were conducted on chocolate agar and semisolid agar using standard methods for both aerobic and anaerobic organisms. The culture results were available before the time of surgery in all study patients. When MRSA culture was positive, mupirocin ointment was used for 3 days. Except for mupirocin in MRSA-positive patients, no additional specific protocols were applied in the patients with positive NCx of microorganisms other than MRSA. Perioperative management was not changed by preoperative NCx.

Patients were advised to shower or bathe with a plain soap on the day before surgery if possible. Preoperative glycemic control was achieved as a glycated hemoglobin value <8.0%. Medical intervention was applied in the patients with glycated hemoglobin values >8.0% to improve glycemic control before surgery.

### Intraoperative Management

Body hair was removed using electric clippers after induction of anesthesia. All operative sites were cleaned and disinfected with chlorhexidine alcohol followed with 10% povidone-iodine. At least 2 minutes later, adhesive iodophor-impregnated plastic incision drapes were applied to the operative field. Surgical hand antisepsis was as follows: the 2-stage or waterless procedure. All staff members of the operating team were wearing 2 pairs of gloves, the outer of which was changed regularly.

Cefazolin was used for antimicrobial prophylaxis against SSI, with an initial dose of 1 g given within 30 minutes of the skin incision and repeated for every 4 hours. For MRSA carriers, 1 dose of 1 g vancomycin was given in the ward and completed within 1 hour before skin incision, followed by a second dose of no more than 4 mg/kg. Antibiotic agents with gram-negative coverage were not administered even in the patients with positive NCx of gram-negative microorganisms before surgery. In all cases, prophylactic antibiotics were continued for no longer than 48 hours.

In coronary artery bypass grafting, we routinely use skeletonized internal thoracic arteries (ITAs), using a harmonic scalpel (Ethicon Endo Surgery, Minneapolis, Minn). Before sternal closure, we removed the bone wax used to the sternal edges was removed as much as possible before sternal closure, because it may increase the risk of infection. The pericardial cavity and mediastinum were washed with 1000 mL normal saline or more. The sternum was reapproximated with 6 stainless steel wires in a simple cerclage fashion. No patients received a topical antibiotic to the cut edge of the sternum. The muscle layers were closed in a simple interrupted technique using 0 coated, braided nylon absorbable sutures. After closing the sternal and muscle layers, the wound was also washed with 500 mL normal saline. Subcutaneous tissue and dermis were closed using 3–0 and 4–0 continuous or intermittent absorbable monofilament sutures, respectively.

### Postoperative Management

The incision was covered with a sterile waterproof transparent dressing with an absorbent pad (Opsite Post-Op Visible; Smith & Nephew, Hull, United Kingdom), a hydrocolloid dressing (Karayahesive; Alcare Co Ltd, Tokyo, Japan), or a sterile liquid adhesive made of octyl-2-cyanoacrylate (Dermabond; Ethicon, Inc, Somerville, NJ). On the seventh postoperative day, the dressing was removed and the patient was permitted to shower.

Blood glucose level was checked repeatedly intra- and postoperatively. If the glucose value was >160 mg/dL, continuous insulin therapy was initiated. Insulin infusion was continued until oral feeding was initiated. When oral feeding was initiated, intravenous insulin was transitioned to a sliding-scale-guided intermittent subcutaneous insulin injection or the oral diabetes medications until the target blood glucose level <150 mg/dL was achieved.

For the surgical patients who needed frequent sputum suction after extubation, we liberally used Portex Mini-Trach (Smiths Medical, Minneapolis, Minn), which is a Seldinger kit for performing a cricothyrotomy, after open heart surgery, even via median sternotomy. It protects the airway for the patients at risk of aspiration, to secure frequent pulmonary toilet. For those who could not be extubated 2 weeks and more after surgery, had copious secretions, became very agitated, and might pull on their endotracheal tube, tracheostomy was indicated to enable long-term mechanical ventilation. Decision for tracheostomy is made collaboratively by representatives of the nursing, cardiothoracic surgery, cardiothoracic anesthesia, and intensivist staff members who believe that it offers several benefits, including frequent and effective pulmonary toilet, reduction in the incidence of ventilator-associated pneumonia reduced sedation requirements, reduction in the work of breathing, and earlier mobilization of the patient that facilitates weaning from mechanical ventilation.^9^

### Definition of SSI

SSIs were defined according to the nosocomial infection surveillance criteria of the Centers for Disease Control and Prevention.^10^ These definitions require signs of infection over the surgical wound, including purulent drainage and an abscess. To determine the pathogens, sampling was done, including deep tissue infected through the wounds of surgical sites, wound debridement of infected tissue, and drainage of infected tissues. Microbiology culture and antibiotic sensitivity or resistance were conducted on chocolate and semisolid agars for both aerobic and anaerobic organisms.

### Statistical Analysis

The normality of distribution of quantitative variables was tested by using the Shapiro-Wilk test. Continuous variables were summarized as mean ± standard deviation or as median (range) according to the normality of the distribution, whereas categorical variables were presented as frequencies and percentages. All pre-, intra-, and postoperative parameters were compared between the groups with and without postoperative SSI. These initial comparisons were conducted by using the χ^2^ test or the Fisher exact test for qualitative variables and the Student *t* test or the Mann-Whitney test for quantitative variables. Moreover, univariate binary logistic regression analysis was performed to investigate the independent associations among pre-, intra-, and postoperative parameters, and postoperative SSI. For the primary outcome, all univariate variables were then entered into a binary logistic multivariate model when the level of *P* in the univariate analysis was < .1. Bootstrapping with 1000 samples was used both in uni- and multivariate logistic regression analyses, and odds ratios (ORs) were reported with corresponding bias-corrected and accelerated 95% confidence intervals (CIs). For internal validation of the multivariate logistic regression model using bootstrapping analysis, the calibration was assessed based on the Hosmer-Lemeshow test and its discrimination ability assessed based on the area under the receiver operating characteristics curve.^11^ A 2-sided *P* value <.05 was considered statistically significant. All statistical analyses were performed with SAS software version 9.3 (SAS Institute Inc, Cary, NC).

## Results

### Patient Demographic Characteristics

A total of 1226 consecutive patients comprised 833 men (68%) and 393 women. The median age was 71 years (range, 19-89 years). Surgery for ischemic heart disease, including isolated coronary artery bypass grafting, was performed in 442 patients (36%), among whom 185 (42%) underwent off-pump coronary artery bypass grafting. Valve and thoracic aortic operations were performed in 435 patients (35%) and 287 patients (23%), respectively. Other operations were performed in 62 patients (5%), including pulmonary endarterectomy, ventricular assist device placement, and surgery for congenital heart disease. The patients who underwent thoracic aortic surgery via lateral thoracotomy were excluded (n = 49). Cardiopulmonary bypass was used in 1045 patients (85%) and not used in 181 patients (15%).

### Preoperative NCx

As shown in Table 1, the nasal swabs from 442 patients (36%) and the pharyngeal swabs in 1115 patients (91%) presented normal flora, respectively. There were 1281 and 127 microorganisms isolated from 658 nasal and 111 pharyngeal specimens, respectively. The most frequent microorganisms were coagulase-negative *Staphylococcus*, followed by *Corynebacterium* spp, *Streptococcus* spp, and MRSA.

**Table 1:** Results of preoperative nasopharyngeal cultures

Variable	Nasal				Pharyngeal			
	All	Postoperative SSI		*P* value	All	Postoperative SSI		*P* value
		Yes (n = 31)	No (n = 1195)			Yes (n = 31)	No (n = 1195)	
Patients								
With normal flora	442 (36)	6 (16)	436 (36)	.02	1115 (91)	17 (55)	1098 (92)	<.001
With abnormal flora	784 (64)	25 (84)	759 (64)		111 (9)	14 (45)	97 (8)	
Isolated microorganisms	1281	28	1253		127	18	109	
Gram-positive cocci								
MSSA	153 (11.9)	3 (10.7)	150 (12.0)	.83	18 (14.2)	2 (11.1)	15 (13.8)	.74
CNS	445 (34.7)	10 (35.7)	435 (34.7)	.91	11 (8.7)	5 (27.8)	9 (8.3)	.69
MRSA	17 (1.3)	2 (7.1)	15 (1.2)	.06	6 (4.7)	2 (11.1)	5 (4.6)	.85
*Streptococcus* spp	169 (13.2)	3 (10.7)	166 (13.2)	.91	19 (15.0)	2 (11.1)	16 (14.7)	.82
*Enterococcus* spp	1 (0.1)	0	1 (0.1)	.88	2 (1.6)	0	2 (1.8)	.56
*Stomatococcus* spp	4 (0.3)	0	4 (0.3)	.76				
Gram-negative rods								
Enterobacter	15 (1.2)	1 (3.6)	14 (1.1)	.75	7 (5.5)	1 (5.6)	6 (5.5)	.99
*Klebsiella* spp	2 (0.2)		2 (0.2)	.83	35 (27.6)		31 (28.4)	.58
*Serratia*	1 (0.1)		1 (0.1)	.88	2 (1.6)		2 (1.8)	.56
*Pseudomonas aeruginosa*	1 (0.1)		1 (0.1)	.88	5 (3.9)	1 (5.6)	5 (4.6)	.35
*Pseudomonas putida*					1 (0.8)		1 (0.9)	.68
*Citrobacter koseri*	2 (0.2)		2 (0.2)	.83				
*Haemophilus influenzae*	5 (0.4)		5 (0.4)	.73	5 (3.9)		5 (4.6)	.85
*Stenotrophomonas spp*	5 (0.4)		5 (0.4)	.73	1 (0.8)		1 (0.9)	.68
*Acinetobacter baumannii*	1 (0.1)		1 (0.1)	.88	1 (0.8)		1 (0.9)	.68
Gram-positive rods								
*Corynebacterium* spp	410 (32.0)	5 (17.9)	405 (32.3)	.15	12 (9.4)	4 (22.2)	8 (7.3)	
*Bacillus*	20 (0.6)	2 (7.1)	18 (1.4)	.10				
Gram-negative cocci								
*Neisseria*	23 (1.8)	1 (3.6)	22 (1.8)	.99	2 (1.6)	0	2 (1.8)	.12
*Moraxella catarrhalis*	7 (0.5)	1 (3.6)	6 (0.5)	.36				

Values are presented as n (%) or n. *SSI*, Surgical site infection; *MSSA*, methicillin-sensitive *Staphylococcus aureus*; *CNS*, coagulase negative *Staphylococcus*; *MRSA*, methicillin-resistant *Staphylococcus aureus*.

### SSI and Pathogens

Postoperative SSI occurred in 31 patients (2.5%), including chest wound SSI in 21 patients (1.7%), and groin or leg SSI related cannulation or saphenous vein harvesting in 10 patients (0.8%). The frequency of SSI varied during the 5-year study period. Table 2 shows the pathogens of these SSIs, whereas negative microbial results were obtained in 8 patients (25.8%) with chest SSI. The most frequent microorganisms were coagulase-negative *Staphylococcus*, MRSA, *Escherichia coli*, and methicillin-sensitive *Staphylococcus aureus*. Coincidence of the pathogens of postoperative SSI with the isolated microorganisms of preoperative NCx were observed in only a few patients with SSI caused by gram-positive bacteria.

**Table 2:** Pathogens of postoperative surgical site infection (SSI)

Pathogen	Chest SSI (n = 21)			Groin and leg SSI (n = 10)		
	n (%)	Coincident with		n (%)	Coincident with	
		Nasal culture (%)	Pharyngeal culture (%)		Nasal culture (%)	Pharyngeal culture (%)
Microbial cultures						
Negative	8 (38)			0 (0)		
Positive	13 (62)			10 (100)		
Isolated microorganisms	18			17		
Gram-positive cocci						
MSSA	1 (6)	0	0	2 (12)	50	0
CNS	5 (28)	40	20	2 (12)	50	0
MRSA	4 (22)	0	25	2 (12)	0	0
*Enterococcus faecium*				2 (12)	0	0
Gram-negative rods						
*Enterobacter cloacae*	1 (6)	0	100	1 (6)		0
*Klebsiella Pneumoniae*	1 (6)	0	0			
*Serratia marcescens*	1 (6)	0	0			
*Pseudomonas aeruginosa*	1 (6)	0	100			
*Citrobacter werkmanii*				1 (6)	0	0
*Escherichia coli*				3 (18)	0	0
*Stenotrophomonas maltophilia*	1 (6)	0	0			
Gram-positive rods						
*Corynebacterium spp*	1 (6)	100	0	3 (18)	66	0
*Propionibacterium acnes*	1 (6)	0	0	1 (6)		0
Nontuberculous mycobacteria						
*Mycobacterium fortuitum*	1 (6)	0	0			

*MSSA*, Methicillin-sensitive *Staphylococcus aureus*; *CNS*, coagulase negative *Staphylococcus*; *MRSA*, methicillin-resistant *Staphylococcus aureus*.

Management of postoperative SSI was not changed by preoperative NCx. For treatment and wound closure in 21 patients with chest SSI, 1 patient (5%) received rectus abdominis myocutaneous flap,^12^ whereas 20 patients (95%) underwent vacuum-assisted closure (VAC) therapy (KCI, West San Antonio, Tex).^13^^,^^14^ The sternal wound was first debrided of foreign material and necrotic tissue and the VAC was then applied. After confirming no pathogens, 4 patients underwent omental flap procedure^15^ with the median duration of VAC of 33 days (range, 14-76 days), whereas other patients (n = 16) underwent simple re-suture in the operating room with the median duration of VAC of 14 days (range, 7-60 days). One patient undergoing omental flap and 1 patient undergoing re-suture died with severe infection 59 and 80 days after surgery, respectively. Both patients experienced poststernotomy MRSA mediastinitis. No patients with groin or leg SSI died after surgery, among whom 8 patients (73%) received VAC therapy, followed by re-suture.

Compared with the patients without SSI, those with SSI had shown significantly higher positive rates of preoperative NCx (nasal: 52% vs 84% [*P* = .02] and pharyngeal: 8% vs 45% [*P* < .001]) (Tables 1 and 3). The sensitivity and specificity of preoperative nasal cultures for postoperative SSI were 81% and 38%, respectively, whereas those of pharyngeal cultures were 45% and 92%, respectively. The positive predictive value of preoperative NCx for ruling out SSI was 3.2% of nasal and 12.6% of pharyngeal, respectively. The negative predictive value of preoperative NCx for ruling out SSI was 98.6% of nasal and 98.4% of pharyngeal, respectively. However, there were no significant differences between the patients with and without SSI in the isolated microorganisms both on nasal and pharyngeal cultures.

**Table 3:** Perioperative variables of the patients with and without postoperative surgical site infection (SSI)

Variable	Patients with SSI (n = 31)	Patients without SSI (n = 1195)	*P* value
Preoperative			
Age (y)	68 ± 9	69 ± 10	.27
Female gender	15 (48)	387 (32)	.040
BMI	24.4 ± 6.0	23.0 ± 3.8	.10
Emergency and urgent	6 (19)	183 (15)	.32
Reoperation	0	47 (4)	.30
Cardiothoracic ratio on chest radiograph (%)	52 ± 5	52 ± 7	.89
Atrial fibrillation	2 (7)	147 (12)	.26
LVEF on echocardiography	54 ± 10	56 ± 11	.15
Hypertension	22 (71)	1003 (82)	.10
Hyperlipidemia	14 (45)	612 (50)	.59
Diabetes mellitus	14 (45)	355 (29)	.044
Treatment			.48
Diet control	3 (22)	143 (12)	
Oral medication	10 (71)	765 (64)	
Insulin	1 (7)	287 (24)	
Glycated hemoglobin (%)	7.1 ± 1.3	6.9 ± 1.1	.45
Smoking	12 (39)	601 (49)	.17
Pulmonary disease	5 (16)	99 (9)	.11
Liver disease	2 (6)	37 (3)	.25
Kidney disease	7 (23)	282 (23)	.58
On hemodialysis	3 (10)	85 (7)	.37
Blood urea nitrogen (mg/dL)	20 ± 9	21 ± 13	.48
Serum creatinine (mg/dL)	1.4 ± 1.7	1.5 ± 2.0	.69
Peripheral arterial disease	6 (19)	355 (29)	.17
Past history of stroke	1 (3)	122 (10)	.17
Past history of cancer	4 (13)	146 (12)	.52
Positive pharyngeal culture	14 (45)	97 (8)	< .001
Positive MRSA	2 (6)	4 (0.3)	.008
Positive nasal culture	26 (71)	759 (64)	.020
Positive MRSA	2 (13)	15 (1)	.007
White blood cell count (/μL)	7025 ± 1540	6229 ± 2328	.030
Hemoglobin (g/dL)	13.2 ± 1.3	12.8 ± 1.9	.22
Platelet count (×10^4^/μL)	21.5 ± 7.4	20.6 ± 7.2	.20
Serum albumin (g/dL)	4.0 ± 0.9	3.9 ± 0.6	.47
Operative			
Surgery for ischemic heart disease	10 (32)	454 (37)	.36
Valve surgery	10 (32)	451 (37)	.37
Thoracic aorta surgery	8 (26)	328 (27)	.54
Operation duration (min)	408 ± 153	391 ± 135	.27
Cardiopulmonary bypass use	24 (77)	1046 (85)	.16
Cardiopulmonary bypass time (min)	205 ± 68	191 ± 76	.13
Cardiac ischemic time (min)	156 ± 56	148 ± 57	.36
Lowest body temperature (°C)	30.6 ± 4.2	30.2 ± 7.9	.22
Circulatory arrest, n (%)	2 (6)	217 (18)	.08
Use of bilateral ITAs	3 (10)	90 (7)	.40
Postoperative			
Intra-aortic balloon pump use	1 (3)	138 (11)	.12
ECMO use	2 (6)	14 (1.1)	.06
Reoperation for bleeding	0	15 (1.2)	.68
Delayed chest closure	1 (3)	10 (0.8)	.24
Portex Mini-Trach∗ or tracheostomy	12 (4/8) (39)	90 (79/11) (8)	.005
In-hospital death	2 (6)	32 (3)	.19

Values are presented as n (%) or mean ± standard deviation. *BMI*, Body mass index; *LVEF*, left ventricular ejection fraction; *MRSA*, methicillin-resistant *Staphylococcus aureus*; *ECMO*, extracorporeal membrane oxygenation; *ITAs*, internal thoracic arteries.

∗ Smiths Medical, Minneapolis, Minn.

### Risk Factors for SSI

As shown in Table 3, significant differences between the patients with and without postoperative SSI were detected in female sex, more prevalence of diabetes mellitus, positive NCx, and higher white blood cell count, as for preoperative variables. Although there were no significant differences in the operative variables, postoperative use of Portex Mini-Trach, tracheostomy, and extracorporeal membrane oxygenation were more frequent in the patients with SSI.

Multivariate analysis identified that independent risk factors for postoperative SSI were female sex (OR, 1.52; 95% CI, 0.38-0.87; *P* = .021), diabetes mellitus (OR, 2.26; 95% CI, 1.89-5.44; *P* = .016), preoperative positive NCx (OR, 1.81; 95% CI, 1.06-1.52; *P* = .034), and postoperative use of Portex Mini-Trach or tracheostomy (OR, 2.57; 95% CI, 1.28-6.37; *P* = .037), as shown in Table 4. The internal validation of the multivariate logistic regression model using bootstrapping analysis showed marginal bias with calibration (Hosmer-Lemeshow test *P* = .482) and discrimination (area under the curve, 0.783).

**Table 4:** Univariate and multivariate predictors for postoperative surgical site infections (SSIs)

Variable	Univariate		Multivariate	
	Adjusted OR (95% CI)	*P* value	Adjusted OR (95% CI)	*P* value
Preoperative				
Age (y)	1.24 (0.82-3.14)	.19		
Female gender	1.28 (1.21-1.69)	.043	1.52 (0.38-0.87)	.02
Body mass index	0.96 (0.93-1.08)	.098	1.03 (0.92-1.23)	.19
Emergency and urgent	0.89 (0.95-1.06)	.13		
LVEF (%)	0.92 (0.90-1.33)	.12		
Diabetes mellitus	1.77 (1.04-2.92)	.022	2.26 (1.89-5.44)	.01
Smoking	1.36 (0.48-3.71)	.21		
Pulmonary disease	0.70 (0.15-3.38)	.36		
Liver disease	0.53 (0.18-1.78)	.23		
Kidney disease	1.92 (0.36-8.17)	.48		
Peripheral arterial disease	1.73 (0.61-4.53)	.22		
Past history of stroke	1.37 (0.88-2.05)	.18		
Positive nasopharyngeal culture	2.12 (1.31-3.46)	.004	1.81 (1.06-1.52)	.02
Positive MRSA	1.84 (1.02-1.47)	.019	1.29 (0.85-1.94)	.23
White blood cell count (/μL)	3.08 (1.77-9.22)	.041	1.07 (0.95-1.86)	.18
Hemoglobin (g/dL)	1.08 (0.94-1.06)	.34		
Serum albumin (g/dL)	1.38 (0.42-4.02)	.53		
Operative				
Surgery for ischemic heart disease	1.61 (0.45-3.12)	.41		
Valve surgery	1.54 (0.49-3.74)	.38		
Thoracic aorta surgery	1.48 (0.35-3.99)	.46		
Operation duration (min)	2.18 (0.44-9.20)	.17		
Cardiopulmonary bypass use	1.49 (0.33-7.15)	.25		
Cardiopulmonary bypass time (min)	2.09 (0.41-7.77)	.15		
Cardiac ischemic time (min)	1.67 (0.58-5.13)	.26		
Circulatory arrest	1.65 (1.10-2.24)	.074	1.19 (0.70-2.21)	.49
Use of bilateral ITAs	1.25 (0.44-3.86)	.57		
Postoperative				
Intra-aortic balloon pump use	0.56 (0.19-1.60)	.27		
ECMO use	1.65 (1.10-2.24)	.053	1.23 (0.86-1.93)	.29
Reoperation for bleeding	1.26 (0.61-1.86)	.65		
Delayed chest closure	1.43 (0.90-2.28)	.33		
Portex Mini-Trach∗ or tracheostomy	2.69 (1.27-10.72)	.018	2.57 (1.28-6.37)	.037

*OR*, Odds ratio; *CI*, confidence interval; *LVEF*, left ventricular ejection fraction; *MRSA*, methicillin-resistant *Staphylococcus aureus*; *ITAs*, internal thoracic arteries; *ECMO*, extracorporeal membrane oxygenation.

∗ Smiths Medical, Minneapolis, Minn.

## Discussion

There are 3 main findings of our study regarding SSI after open heart surgery via median sternotomy:

• Postoperative SSI still occurred in 2.5% of our patients who cared with the standard SSI bundle at our institute due to coagulase-negative *Staphylococcus*, MRSA, and *E coli*, with mortality of 6.5%.
• Significant coincidence of the SSI pathogens with the NCx microorganisms was not observed.
• Positive preoperative nasopharyngeal culture was an independent predictor of postoperative SSI, as well as female sex, diabetes mellitus, and postoperative use of Portex Mini-Trach or tracheostomy.

Because nasal carriage of MRSA increases the risk of SSI in cardiac surgery,^16^ the guidelines and expert consensus documents5-7 recommend nasal screening and mupirocin decolonization for the patients with MRSA, although several studies have found no effect of mupirocin treatment in the incidence of SSI. We found that patients with SSI showed significantly higher positive rates of NCx compared with patients without SSI. This finding may provide additional significance to the preoperative NCx screening for not only MRSA but also other microorganisms to predict postoperative SSI. As shown in Table 2, there is diverse bacterial pathogens distribution in our patients with SSI. An important finding was that the positive NCx predisposed patients to SSI but not necessarily with MRSA. Although gram-positive bacteria are the main bacteria implicated, gram-negative bacilli were also involved, as previously reported,^17^^,^^18^ which should not be present in the air of the operating room and do not generally reside on the skin of the chest. The patients with positive preoperative NCx are likely to have altered vital environments with altered intestinal flora and susceptibility to antibiotics. Gram-negative bacilli for SSI may be partly ascribable to preoperative decolonization of nasal MRSA carriage, possibly replacing it. We therefore recommend combined use of nasal swabs with high sensitivity and pharyngeal swabs with high specificity, as shown in the present study, for not only MRSA but also for other microorganisms.

More strict SSI bundles are recommended in the patients with positive preoperative NCx, whether MRSA-positive or not. Preoperative decontamination of the nasopharynx and oropharynx with chlorhexidine gluconate, as previously reported, should be included.^19^^,^^20^ It may be beneficial to add the perioperative antibiotics with gram-negative coverage to prophylactic use of cefazolin and/or vancomycin in the patients with positive NCx of gram-negative microorganisms. In such high-risk patients with positive preoperative NCx, it may be also beneficial to use the prophylactic negative pressure wound therapy (VAC therapy), as recommended by Tabley and colleagues.^21^ Furthermore, when we encounter the signs of postoperative SSI, we would refer to our results of SSI pathogens in the patients with positive preoperative NCx of the present study.

Numerous risk factors of postoperative SSI have been identified, including preoperative variables such as age, female sex, smoking status, obesity, diabetes, hypertension, chronic lung disease, peripheral vascular disease, kidney disease, immunosuppressive treatment, and ejection fraction; operative factors such as resternotomy, bilateral ITAs harvest, prolonged bypass and operative times, and intraoperative blood products; and postoperative variables such as respiratory failure and tracheostomy.^22^ Although our results likely coincided with the previous studies, we did not investigate immunosuppressive treatment and intraoperative blood products. We showed no differences regarding age, obesity, cardiac function, lung, vascular, and kidney disease, bilateral ITAs, and procedure time in this study, probably due to the population difference.

Among our noteworthy results was the use of Portex Mini-Trach or tracheostomy as a risk factor of SSI. Previous studies also demonstrated that tracheostomy was an independent risk factor for poststernotomy SSI.^23^^,^^24^ We included less invasive use of the Portex Mini-Trach in addition to tracheostomy as a means of facilitated respiratory management following surgery. The spread of bacteria from the tracheostomy site to the sterile unhealed sternotomy has not been well addressed. Respiratory failure per se, rather than tracheostomy, may be associated with an increased risk of SSI in high-risk patients with complicated postoperative recovery, as Rahmanian and colleagues^9^ reported. However, based on our results, our liberal use of tracheostomies cannot be considered a best practice. Therefore, we plan to curtail the use of tracheostomies for postoperative patients. The second noteworthy result was that preoperative leukocytosis may be closely related to SSI, as reported in recent studies.^25^^,^^26^ As Strobel and colleagues suggested,^27^ preoperative leukocytosis may be an immune response to preexisting pathogens associated with preoperative positive NCx, as demonstrated in our study. The third noteworthy result was that a delayed chest closure is not a risk factor for SSI, although previous studies have been controversial on this topic.^28^^,^^29^

Our study is mainly limited by its retrospective design, the relatively small size of the study population, and wide variety of operative procedures performed. This study aimed to assess roughly the risk of SSI in real-world patients undergoing cardiac and thoracic aortic surgery rather than detailed surgical categories. Therefore, we investigated consecutive patients to eliminate the biases of heterogeneous demographic characteristics of study patients. In addition, although all patients included for analysis were cared for by a multidisciplinary team, according to our institutional standards, it is unclear whether our results can be readily reproduced in other institutions. The incidence of chest wound SSI (1.7%) in our study is 3 to 4 times higher than that in the most recent Society of Thoracic Surgeons Database report.^4^ This may be due to our incomplete adherence to guidelines^5^, ^6^, ^7^ regarding the duration of prophylactic mupirocin ointments, additional use of aminoglycoside with prophylactic vancomycin, and use of bone wax for the sternum. Complete adherence to the guidelines may have yielded different results. Lastly, the surgical team was not swabbed for infection control in this study. The reported MRSA carriage rates in the health care workers were 23.7%.^30^ Although most studies and guidelines focus on infection control and MRSA decolonization in patients rather than considering health care providers as the source, we should probably involve routine systematic screening and decolonization of health care workers to decrease postoperative SSI.

## Conclusions

Although preoperative NCx have been used for MRSA screening, the positive NCx for microorganisms other than MRSA may also be useful to predict SSI after cardiac and thoracic aortic surgery via median sternotomy.

### Conflict of Interest Statement

The authors reported no conflicts of interest.

The *Journal* policy requires editors and reviewers to disclose conflicts of interest and to decline handling or reviewing manuscripts for which they may have a conflict of interest. The editors and reviewers of this article have no conflicts of interest.
